# Rider Variables Affecting the Stirrup Directional Force Asymmetry during Simulated Riding Trot

**DOI:** 10.3390/ani12233364

**Published:** 2022-11-30

**Authors:** Paolo Baragli, Alberto Alessi, Marco Pagliai, Martina Felici, Asahi Ogi, Lesley Hawson, Angelo Gazzano, Barbara Padalino

**Affiliations:** 1 Department of Veterinary Sciences, University of Pisa, 56124 Pisa, Italy; 2 Research Center “E. Piaggio”, University of Pisa, 56122 Pisa, Italy; 3 Addestramento Etologico, 51028 Pistoia, Italy; 4 Department of Agricultural and Food Sciences, University of Bologna, 40127 Bologna, Italy; 5 Harness Racing Victoria, Flemington, VIC 3031, Australia

**Keywords:** equitation, rider, riding style, rising trot, horse, welfare

## Abstract

**Simple Summary:**

Preventing possible pain caused by functional asymmetry of both rider and horse needs an investigation of the rider-related variables that could affect the distribution of mass on the stirrups. Using digital load cells applied to the stirrup leathers of a wooden horseback model, we measured the directional force exerted by both legs of 147 riders on stirrup straps determining possible asymmetries. Furthermore, we investigated the possible associations between the asymmetry and eight variables related to the riders: gender, age, level of riding ability, years of riding experience, riding style, motivation of riding, primary discipline, and handedness. The preliminary findings confirmed that the majority of the riders are asymmetrical while riding on wooden horseback. Riding style seems to be a risk factor for asymmetry. Further studies using larger sample groups controlled for leg dominance and riding experience are required.

**Abstract:**

Riders’ asymmetry may cause back pain in both human and equine athletes. This pilot study aimed at documenting in a simple and quick way asymmetry in riders during a simulation of three different riding positions on wooden horseback using load cells applied on the stirrup leathers and identifying possible associations between riders’ asymmetry and their gender, age, level of riding ability, years of riding experience, riding style, motivation of riding, primary discipline and handedness. After completing an interview to obtain the previously mentioned information, 147 riders performed a standardized test on a saddle fixed on a wooden horseback-shaped model. The riding simulation was split into three phases of 1 min each: (1) sit in the saddle, (2) standing in the stirrups and (3) rising trot. The directional force on the left and the right stirrup leathers was recorded every 0.2 s. A paired t-test was performed on the recorded data to test the difference (i.e., asymmetry) in each phase. In phases 1, 2 and 3, 99.3% (53.4% heavier on the right (R)), 98% (52.8% heavier on the left (L)) and 46.3% (51.5% heavier on the left (L)) of the riders were asymmetrical, respectively. Chi-square tests showed a significant association between riding ability and riding experience, but no significant association between reported handedness and calculated leg-sidedness (*p* > 0.05). Univariate logistic (1: asymmetry, 0: symmetry) regression analysis was performed only on the phase 3 data. One-hand riders were found twice more likely to be asymmetrical than two-hand riders (Odds Ratio (OR): 2.18, Confidence Interval (CI): 1.1–4.29; *p* = 0.024). This preliminary study confirmed that the majority of the riders are asymmetrical in load distribution on stirrups and suggested the riding style as a possible risk factor for asymmetry.

## 1. Introduction

Equestrianism is a unique sport. It involves two athletes of different species and morphology, moving together as a pair [1]. In equestrianism, asymmetry has been defined as an uneven forces’ distribution in the kinetics of the horse rider dyad [2]. It has been reported that asymmetry and functional laterality in the body movements may be related to characteristics of both humans and horses [3]. The laterality—the preference most mammals show for one side of their body over the other—is strongly task-dependent [4]. In humans, laterality is more pronounced in the upper limbs, especially for accurate gestures, but it was found and described also for the lower limbs through, for example, an ad hoc walking test [5]. While the laterality of the horse and the rider could contribute to potential asymmetry [2], as it is difficult to measure the force exchange at the interface between horse and rider [6]. Moreover, asymmetry could be due to the rider, the horse, the combination of rider–horse and/or the type of equipment being employed [2]. Variables, such rider’s experience, riding position, type of gait, anatomical and functional characteristics of both rider and horse, and saddle design and position have been identified as contributing to degree of asymmetry found in rider–horse movement [1,6,7,8,9,10].

Previous studies have used video recording, pressure measuring saddle mats and accelerometers [2,3,8,9] to measure how the rider’s body can influence the range of motion of the horse’s thoracolumbar spine and gait quality [2,9,10,11]. The changes horse movement created by the rider can influence horse performance and even long-term soundness [3]. The rider’s legs wrap around the horse’s thorax are used to apply cues usually associated with acceleration and/or direction [12]. It is likely that the rider’s legs also play a role in helping the rider to maintain alignment with the horse’s center of mass and, therefore, play an important role in rider–horse biomechanics. In modern day equestrianism, it is usual for riders to use stirrups to assist with their balance by attenuating the acceleration created by the horse’s movement [13].

The rider’s interaction with the stirrup has been shown to impact on the horse and the rider–horse dyad. The load that a rider applies on the stirrups can change the load measured under the saddle [11]. Higher forces are applied on the stirrups in the standing phase of rising trot [7,11], while shortening a single stirrup has been shown to induce sufficient asymmetry in the force distribution to influence the horse’s kinematics and fetlock range of motion [10]. Leg length inequality is a common cause of gait asymmetry in humans [14]. Mackechnie-Guire and colleagues [10] recently reported that, in the case of anatomical finding of legs of different length, the rider tends to tilt their trunk toward the short leg. Hypothesizing that many riders would be asymmetric, the aims of the study were to quickly document the prevalence of riders’ asymmetry measured using cheap and easy-to-use load cells applied to the stirrup leathers while sitting in the saddle, standing in the stirrup, and simulating rising trot and to investigate possible associations between observed asymmetry and individual rider characteristics. Load cells that detect directional force were used to measure the mass riders were distributing onto each stirrup.

## 2. Materials and Methods

### 2.1. Sample

Data were collected from 147 riders recruited voluntarily at the Fiercavalli horse show (www.fieracavalli.it, accessed on 23 October 2022, Verona, Italy) in November 2017. Each rider was interviewed by one of the authors to collect information about: gender, age, level of riding ability, years of riding experience, riding style, motivation of riding, primary discipline, and handedness—see Table 1 for details. The median of the riders’ age was 37 (Interquartile Range (IQR): 27–78), with the youngest rider of 18 years and the oldest of 74 years of age.

### 2.2. Saddle Test

**Figure 1:**
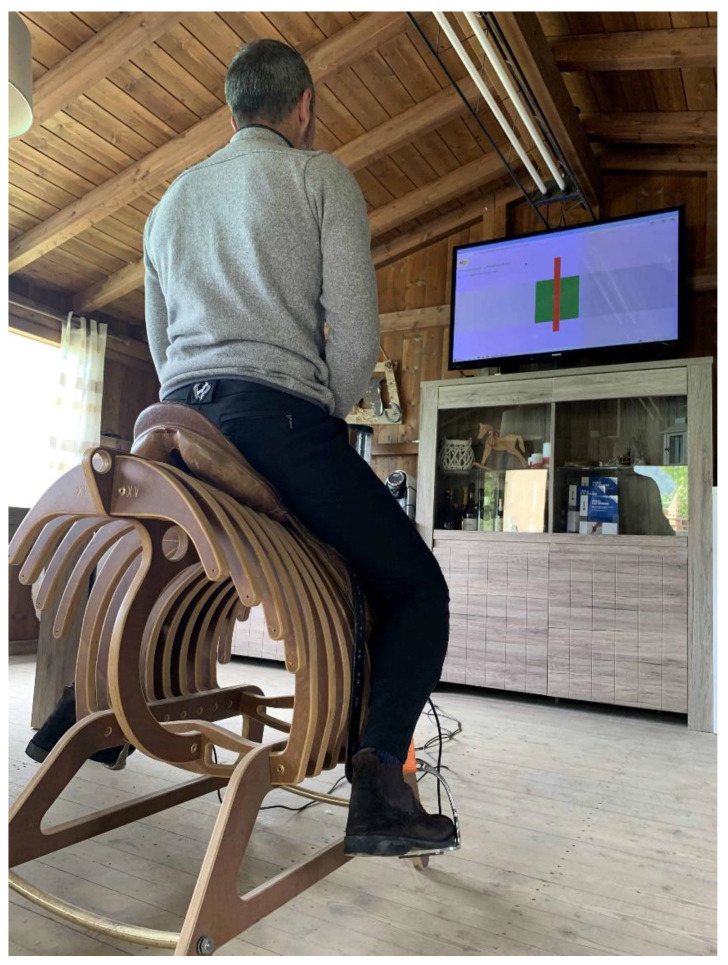
The wooden horse back.

After having responded to the interview, each volunteer rider had the opportunity to practice on the wooden horseback before performing official test, for as long as each of them deemed necessary. Each rider had also the opportunity to adjust the length of the stirrups according to his/her habit and comfort. Riders could assume the feet position within the stirrups that was most congenial to them. As the volunteer riders were visiting Fieracavalli, no standardized footwear was worn during the tests. After preparation, the riders performed a standardized test on a saddle fixed on a wooden horseback shape (Figure 1). The test was split into three phases of 1 min each: (1) sit on the saddle, (2) standing up in the stirrups, (3) simulated rising trot. During the test, the riders were asked to keep their hands on the hips, to maintain a neutral standardized position without affecting the distribution of the mass on stirrups during those simulated riding activities.

**Figure 2:**
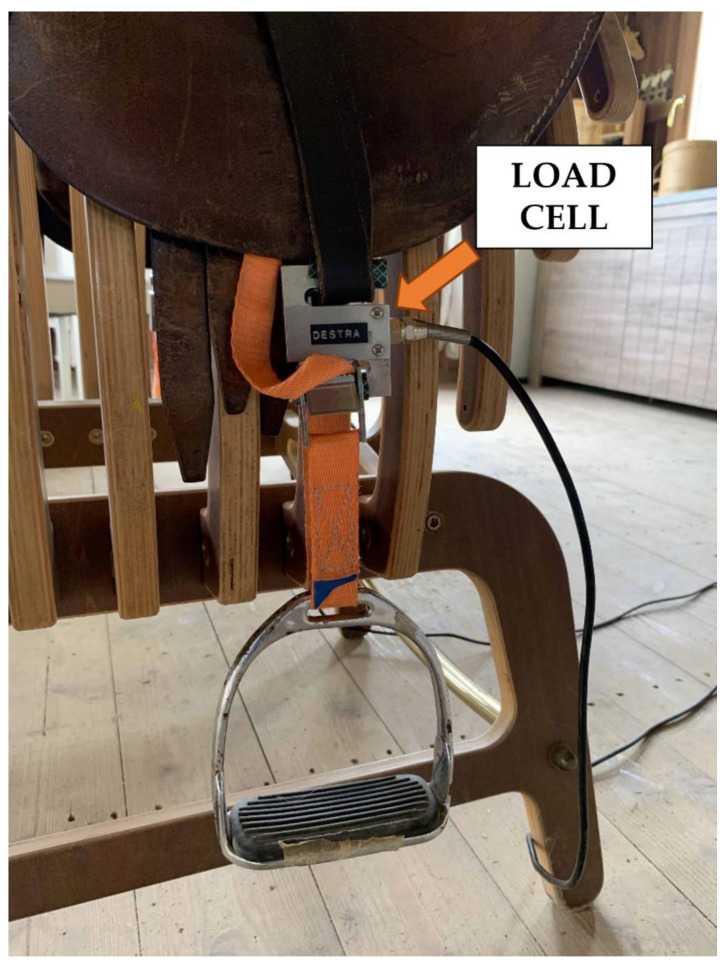
The stirrup leathers equipped with the load cell and the load cell amplifier.

Each stirrup leather of the saddle was equipped with a hand-crafted directional force measuring device. This device was assembled with components bought online (Figure 2). For each stirrup, the device included a load cell CZL301C (Phidgets Inc., Calgary, AB, Canada) and a load cell amplifier HX711 (Avia Semiconductor Ltd., Xiamen, China). The device of both stirrups sent collected data to the hardware Raspberry PI 3 Model B (Raspberry Foundation, Cambridge, UK), which collected the signal through dedicated software. The equipment recorded the directional force produced by the rider’s leg on the left and right stirrup every 0.2 s. The directional force detected by the load cell is a proxy for the mass the rider is distributing to each stirrup via the Newton’s Second Law of Motion; F = m × a, where F = Force, m = mass and a = acceleration.

### 2.3. Statistical Analysis

The raw data registered during each test were subjected to descriptive statistics. Then, to identify whether a rider was asymmetric, a paired t-test was performed on the recorded raw data registered by the right and left stirrup sensor during each test, namely, (1) sit on the saddle, (2) standing up in the stirrups, (3) simulated rising trot. Chi-square tests were performed to identify possible associations between handedness (Left (L), Right (R), Ambidextrous) and leg sidedness (L, R), and between riding ability (beginner, intermediate, advanced, professional) and riding experience (1–5 years, 6–10 years, 11–15 years, 16–20 years, 20+ years). Frequency tables were then realized to report the percentage of asymmetry in each test, and symmetry was more than 5% of the sample only in the simulated trot. Consequently, only for phase 3 data, a frequency table of the explanatory variable was calculated and univariate logistic (1: asymmetry, 0: symmetry) regression analysis was performed using the following predictive variables collected through the interview: rider’s gender, age, level of riding ability, years of riding experience, riding style, motivation of riding, primary discipline, and handedness. *p*-value was set at 0.05. The analysis was performed using GenStat (20th edition, VSN International Ltd., England, UK).

## 3. Results

The directional force exerted by both left and right leg was registered by the load cells on the stirrup leathers. An example of the descriptive statistics of directional force collected during the three tests performed by one of the 147 riders is shown in Table 2, while the descriptive statistics of riders are reported in Table 3. Table 4 shows the descriptive statistics based on the type of riding style (one-hand vs two-hands) in each phase of the test.

During phase 1, 146/147 (99.3%) riders were asymmetrical, and among them, 78/146 (53.4%) were heavier on the right (R) and 68/146 (46.6%) were heavier on the left. During phase 2, 144/147 (98%) riders were asymmetrical, and among them, 68/144 (47.2%) were heavier on the right (R) and 76/144 (52.8%) were heavier on the left (L). During phase 3, 68/147 (46.3%) were asymmetrical, and among them, 33/68 (48.5%) were heavier on the right (R) and 35/68 (51.5%) were heavier on the left (L).

Almost one-third of riders were consistently heavier with the same leg, the other two-third changed the heavier leg during the three phases. Only 66 riders were asymmetrical in all three phases, and of those 26 were consistently heavier on the same legs (L or R), while the remaining forty riders changed to the being heavier on the opposite leg within the three phases.

**Figure 3:**
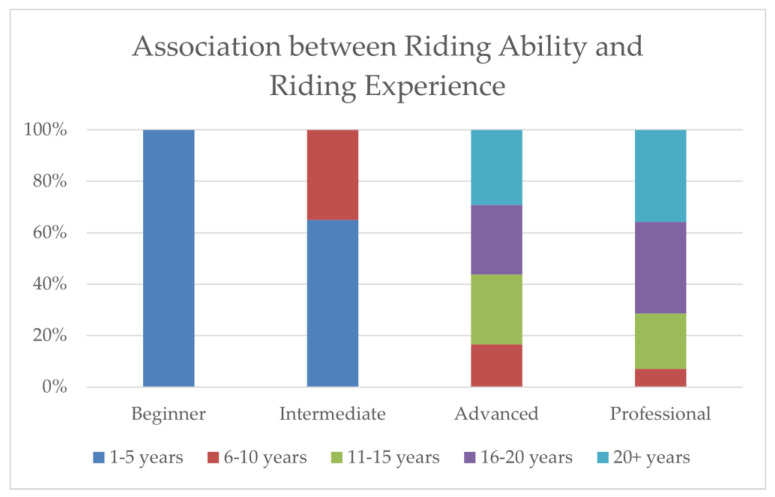
Association between riding ability and riding experience of the 147 volunteer riders who performed the simulation riding tests on a wooden horse back.

Considering all the riders (*n* = 147), as expected, there was an association between level of riding ability and years of riding experience (χ^2^ = 21.74; *p* < 0.001) (Figure 3). However, there was no association between the nature of handedness (right, left, both hands) and the heavier leg during: (1) sit on the saddle test (χ^2^ = 1.66; *p* = 0.437), (2) standing up in the stirrups test (χ^2^ = 1.96; *p* = 0.376) and 3) simulated rising trot test (χ^2^ = 0.92; *p* = 0.630). Considering only the asymmetrical riders in all three phases (*n* = 66), there was no association between the handedness and the heavier leg during test 1 (χ^2^ = 1.72; *p* = 0.423), test 2 (χ^2^ = 2.00; *p* = 0.367) and test 3 (χ^2^ = 0.40; *p* = 0.817).

The characteristics of the asymmetrical riders during rising trot (*n* = 68) (i.e., Test 3) are reported in Table 5. Univariate logistic regression revealed that riders using one hand to ride a horse, as a riding style, were twice as likely to be asymmetrical than two hand riders (Odds Ratio (OR): 2.18, Confidence Interval (CI): 1.10–4.29; *p* = 0.024) during simulated rising trot on a fixed saddle. No other significant associations were found (Age, *p* = 1.000; Discipline, *p* = 0.315; Experience, *p* = 0.578; Handedness, *p* = 0.937; Riding level, *p* = 0.493; Riding purpose, *p* = 0.625).

## 4. Discussion

Using a cheap and easy-to-use load cell, we investigated in a quick way the directional force that the riders exert on the stirrups during three simulated riding positions (sit, stand and rising trot) on a stationary horse model. Possible associations between the rider’s asymmetry and rider’s background were also explored. As hypothesized, most of the riders were asymmetrical, as they distribute a different force and mass on the two stirrups. Since our design excluded the horse as a variable (i.e., riders were tested on saddle fixed on a wooden horseback), the asymmetry detected was exclusively due to the rider. For all three riding positions, the results showed that the legs’ load was different between left and right. The load on the stirrups varied depending on the position (sit, standing and rising trot) and mass distribution often shifted from one leg to the other during the three testing phases. Few riders demonstrated same leg mass distribution in all three phases. Only the riding style, as defined by the use of one or two hands on the reins, had a significant association with increased asymmetry of mass in the stirrups. Our quick test and its preliminary data may be useful to predict asymmetry in riders and possible back pain in horses.

In our study, riders showed great variability in the load recorded on the stirrups during each test, and this caused a significant amount of standard deviation. This may be due to the overall directional force calculation for the entire population and for all the variables examined. It is also worth noting that during the rising phases (phases 2 and 3) the standard deviation values are higher than in phase 1. It is, therefore, possible that while standing on stirrups, some riders lost balance or do not put enough force on the stirrup, thus balancing in another point of the wooden horseback. Moreover, during rising trot alternated sitting position (less load on the stirrups) and rising positions (more load on the stirrups), and this alternation of loading and unloading the weight on the stirrups may have contributed to the large variability recorded in phase 3 of the test. At the time of test, riders did not wear standardized footwear, did not assume standardized foot positions, and did not have standardized stirrups lengths, and this may have influenced a different assumption of posture and, consequently, load on the stirrups, causing elevated variation in the recorded values. This finding should be confirmed on a riding test using the same technology

Although it has been established that riders can influence force distribution impacting on the horse’s back [9,15], few studies have investigated the variation of leg mass distribution in riders between different riding positions. Symes and Ellis [6] claimed that asymmetry is linked with the difference in length of the rider’s legs affecting the biomechanics of the whole body of the rider. Gandy et al. [16] found greater hip rotation on the right side in 83% of riders tested led to less mobility of the pelvis; on the contrary, we did not find a consistent pattern of asymmetrical force distribution in the stirrups for all riding positions for most of the riders. This may be because our measurements were carried out as a simulation on a saddle fixed on a wooden horseback; instead, most of the studies conducted about the rider–horse kinematics have been performed on real horses [8]. The absence of a real horse and its movement could have reduced the effect linked to the variable “experience” in the distribution of the load.

While the present study confirmed previous research that the load on the stirrups is greater in standing and in standing phase of the rising trot compared to sitting in the saddle [11], we did not find evidence that asymmetry reduced with riding experience. Clayton and Hobbs [1] reported that right-handed riders often present a right pelvic rotation and left trunk rotation leading to a “collapsed” hip that contributes to a higher load on the right side of the saddle. Most of our test riders were right-handed, but we did not find any association between handedness and asymmetry of leg load. However, our data may be limited by the low number of left-handed riders tested and warrants further investigation.

In our study, the riding ability and experience resulted associated. This was expected because usually riders who have been riding for more years are also those who then engage in more advanced levels of riding [7]. However, it is worth noting that we were not able to assess the riding ability since it was only a simulation, and this information was recalled and self-reported, which is usually considered typical limitations of surveys and interviews [17,18]. Considering that this information was self-reported, our findings reflets the genuinely of the information collected by the volunteer data. It is also worth noting that there was a real interest in the riders to test their symmetry, even if there were many more attractions at the horse show. This highlights the growing interest in riders in horse welfare [18] and their will to improve their ability to enhance the health of their horses.

Riding style (defined as one hand holding the reins compared to holding a rein in each hand) did affect the rider’s leg load asymmetry. This may be due to both the position of the hands, but also the different position of the feet and the different habit of assuming certain riding positions while riding. It is worth noting that, in our test, it was required to perform rising trot to one-handed riders, who are not usually accustomed to it. This may have challenged their balance and, therefore, their symmetry and may have biased our results. However, our findings are in line with the literature and riding style should be listed as a risk factor for asymmetry and subsequent back problems in both riders and horses [1,7]. Limiting one-handed riding of horses may assist in reducing the incidence of back pain in both horses and riders. That said, the present findings must be interpreted with caution because our study has several limitations: the absence of a real horse, the imbalance of handedness amongst the limited number of participants, the lack of detection of riders’ weight, leg sidedness and leg lengths. Another important limitation was the duration test, which was set to 1 min. However, it is worth noting to emphasize the context and aim of the present investigation. In our study, asymmetry detection was intended to be quick, simple, without disturbing the fair visitors. A simple and cheap load cell affixed to the stirrups leathers was employed, instead of expensive (e.g., saddle pads) or time-consuming (e.g., video recording) methods, and we proved its effectiveness in detecting asymmetry in a general rider population. Overall, the use of inexpensive, easy-to-use and quick-to-assemble instrumentation allowed an extensive data collection, even in the context of fairs dedicated to riding enthusiasts, suggesting that this technology could be used as a preliminary cheap, easy way to identify asymmetries in riders that does not involve videos or expensive saddle pads test for determining rider’s asymmetry. Further studies using a larger sample size with equal numbers of left and right-legged riders, with both riding styles, over a longer test and comparing the measurements of load cells in different parts of the stirrups (e.g., stirrup pads) are, however, needed to confirm our preliminary findings.

## 5. Conclusions

As expected, almost all the riders tested were asymmetrical while sitting on the saddle or standing in the stirrups. The incidence of the asymmetric loading of stirrups decreased in rising trot in 54% of the riders. Riders who performed working riding (one hand) were two times more likely to be asymmetrical in stirrup loading compared to English style riders (two hand). This association between asymmetry and riding style may be an important consideration for both riders who experience back pain from riding and clinicians treating horses for back problems, especially during ridden rehabilitation. Our pilot study suggests a cheap and easy way for determining riders’ asymmetry but further research to identify the incidence of asymmetries in stirrup loading and the impact such asymmetries have on both the performance and health of both the horse and rider is required.

## Figures and Tables

**Table 1:** Explanatory variables. Categories created from the responses to the interview’s questions of 147 riders.

Explanatory Variables	Categories
Gender (*n*/%)	Females 77/147 (52.4%) Males 70/147 (47.6%)
Mean age (± SD)	37.8 ± 12.4
Level of riding ability ^1^ (*n*/%)	Beginner 27/147 (18.4%) Intermediate 28/147 (19%) Advanced 71/147 (48.3%) Professional 21/147 (14.3%)
Years of riding experience ^2^ (*n*/%)	1—45/147 (30.6%) 2—23/147 (15.6%) 3—23/147 (15.6%) 4—27/147 (18.4%) 5—29/147 (19.8%)
Riding style ^3^ (*n*/%)	Working riding 87/147 (59.2%) English riding 60/147 (40.8%)
Motivation of riding ^4^ (*n*/%)	Leisure 103/147 (70.1%) Competitive 44/147 (29.9%)
Discipline ^5^ (*n*/%)	Show jumping 11/147 (7.5%) Flatwork 42/147 (28.6%) Western 20/147 (13.6%) Hacking 59/147 (40.1%) Other 15/147 (10.2%)
Handedness (*n*/%)	Right 127/147 (86.4%) Left 8/147 (5.4%) Both 12/147 (8.2%)

^1^ Level of riding ability was self-reported and based on the following criteria: beginner (the respondent was still taking riding lessons), intermediate (respondent was able to ride without the rider coach), advanced (respondent was competing at an amateur level), professional (respondent was paid for riding). ^2^ Years of riding experience refers to the years of equestrian activity. Five categories have been defined: 1 (from 1 to 5 years of activity), 2 (from 6 to 10 years), 3 (from 11 to 15 years), 4 (from 16 to 20 years) and 5 (more than 20 years). ^3^ Riding style refers to the riding type and was divided in two categories: working riding (when the horse is driven using one hand alone, such as western style but not limited to) and English riding (employed in equestrian Olympic disciplines in which the horse is driven using both hands). ^4^ Information about the motivation of riding (leisure or competitive purpose) has been also collected. ^5^ All volunteers were also divided based on the type of discipline carried out with the horse. Five categories have been defined: 1 (show jumping), 2 (flatwork), 3 (western or similar), 4 (hacking) and 5 (other including dressage).

**Table 2:** Descriptive statistics of the directional force (mN/cm^2^) registered during the test from the left (L) and right (R) leg of one of the 147 volunteer riders.

Parameter	Phase 1 (L)	Phase 1 (R)	Phase 2 (L)	Phase 2 (R)	Phase 3 (L)	Phase 3 (R)
Mean	674.72	820.54	2468.11	2583.62	1709.42	1746.13
SD	43.96	51.18	38.49	32.55	656.75	670.52
min	452.65	633.11	2345.01	2485.80	390.04	399.55
max	741.91	911.47	2525.11	2663.06	2608.00	2527.41
Median	674.26	810.11	2475.22	2587.17	2042.71	2059.43

Phase 1: sit on the saddle; Phase 2: standing up on the stirrups; Phase 3 simulated rising trot.

**Table 3:** Descriptive statistics of the directional force (mN/cm^2^) registered during the test from the left (L) and right (R) leg.

Parameter	Phase 1 (L)	Phase 1 (R)	Phase 2 (L)	Phase 2 (R)	Phase 3 (L)	Phase 3 (R)
Mean	655.08	658.26	2044.30	2038.20	1409.27	1409.73
SD	255.33	250.40	488.40	468.26	684.66	667.20
min	20.14	4.31	213.19	154.96	24.60	58.70
max	1468.13	1801.74	4646.87	4520.36	3952.98	4066.92
Median	609.22	598.29	1948.48	1960.61	1427.75	1422.69

Phase 1: sit on the saddle; Phase 2: standing up on the stirrups; Phase 3 simulated rising trot.

**Table 4:** Descriptive statistics of the directional force (mN/cm^2^) registered during the phase 3 from the left (L) and right (R) leg for English riding two-hand riders and working riding one-hand riders styles.

Parameter	Phase 1 (L)		Phase 1 (R)		Phase 2 (L)		Phase 2 (R)		Phase 3 (L)		Phase 3 (R)	
	ER	WR	ER	WR	ER	WR	ER	WR	ER	WR	ER	WR
Mean	597.32	696.24	608.11	694.01	1943.32	2116.28	1955.34	2097.25	1340.88	1458.04	1348.39	1453.45
SD	200.69	280.86	209.05	270.51	423.19	518.15	424.80	488.43	637.11	712.67	631.18	688.43
min	20.14	211.75	4.31	247.68	422.61	213.19	436.23	154.96	80.13	24.6	81.85	58.70
max	1445.18	1468.13	1801.74	1625.49	3148.86	4646.87	3117.67	4520.36	3224.27	3952.98	3115.39	4066.92
Median	599.53	618.355	582.91	627.87	1851.92	2006.45	1814.24	2957.27	1397.55	1454.60	1383.90	1456.41

Phase 1: sit on the saddle; Phase 2: standing up on the stirrups; Phase 3 simulated rising trot. ER: English riding; WR: Working riding.

**Table 5:** Frequency table of the explanatory variable related to the riders during rising trot on a wooden horseback (Test 3).

Explanatory Variables	Asymmetrical Riders	Symmetrical Riders
Gender (*n*/%)	Females 30/68 (44.1%) Males 38/68 (55.9%)	Females 47/79 (59.5%) Males 32/79 (40.5%)
Mean age (±SD)	39.7 ± 12.3	37.1 ± 12.5
Level of riding ability ^1^ (*n*/%)	Beginner 16/68 (23.5%) Intermediate 12/68 (17.7%) Advanced 30/68 (44.1%) Professional 10/68 (14.7%)	Beginner 11/79 (13.9%) Intermediate 16/79 (20.3%) Advanced 41/79 (51.9%) Professional 11/79 (13.9%)
Years of riding experience ^2^ (*n*/%)	1—25/68 (36.8%) 2—10/68 (14.7%) 3—8/68 (11.8%) 4—12/68 (17.7%) 5—13/68 (19.1%)	1—20/79 (25.3%) 2—13/79 (16.5%) 3—15/79 (19%) 4—15/79 (19%) 5—16/79 (20.3%)
Riding style ^3^ (*n*/%)	Working riding 47/68 (69.1%) English riding 21/68 (30.9%)	Working riding 40/79 (50.6%) English riding 39/79 (49.4%)
Motivation of riding ^4^ (*n*/%)	Leisure 49/68 (72.1%) Competitive 19/68 (27.9%)	Leisure 54/79 (68.4%) Competitive 25/79 (31.6%)
Discipline ^5^ (*n*/%)	Show jumping 3/68 (4.4%) Flatwork 21/68 (30.9%) Western 11/68 (16.2%) Hacking 29/68 (42.7%) Other 4/68 (5.9%)	Show jumping 8/79 (10.1%) Flatwork 21/79 (26.6%) Western 9/79 (11.4%) Hacking 30/79 (38%) Other 11/79 (13.9%)
Handedness (*n*/%)	Right 58/68 (85.3%) Left 4/68 (5.9%) Both 6/68 (8.8%)	Right 69/79 (87.3%) Left 4/79 (5.1%) Both 6/79 (7.6%)

^1^ Level of riding ability was self-reported and based on the following criteria: beginner (the respondent was still taking riding lessons), intermediate (respondent was able to ride without the rider coach), advanced (respondent was competing at an amateur level), professional (respondent was paid for riding). ^2^ Years of riding experience refers to the years of equestrian activity. Five categories have been defined: 1 (from 1 to 5 years of activity), 2 (from 6 to 10 years), 3 (from 11 to 15 years), 4 (from 16 to 20 years) and 5 (more than 20 years). ^3^ Riding style refers to the riding type and was divided in two categories: working riding (when the horse is driven using one hand alone, such as western style but not limited to) and English riding (employed in equestrian Olympic disciplines in which the horse is driven using both hands). ^4^ Information about the motivation of riding (leisure or competitive purpose) has been also collected. ^5^ All volunteers were also divided based on the type of discipline carried out with the horse. Five categories have been defined: 1 (show jumping), 2 (flatwork), 3 (western or similar), 4 (hacking) and 5 (other including dressage).

## Data Availability

The data presented in this study are available on request from the corresponding author.
